# Forecasting HFMD Cases Using Weather Variables and Google Search Queries in Sabah, Malaysia

**DOI:** 10.3390/ijerph192416880

**Published:** 2022-12-15

**Authors:** Vivek Jason Jayaraj, Victor Chee Wai Hoe

**Affiliations:** 1 Department of Social and Preventive Medicine, Faculty of Medicine, Universiti Malaya, Kuala Lumpur 50603, Malaysia; 2 Ministry of Health Malaysia, Putrajaya 62000, Malaysia

**Keywords:** coxsackie, EV71, prediction model, meteorology, Google trends, ARIMA

## Abstract

HFMD is a viral-mediated infectious illness of increasing public health importance. This study aimed to develop a forecasting tool utilizing climatic predictors and internet search queries for informing preventive strategies in Sabah, Malaysia. HFMD case data from the Sabah State Health Department, climatic predictors from the Malaysia Meteorological Department, and Google search trends from the Google trends platform between the years 2010–2018 were utilized. Cross-correlations were estimated in building a seasonal auto-regressive moving average (SARIMA) model with external regressors, directed by measuring the model fit. The selected variables were then validated using test data utilizing validation metrics such as the mean average percentage error (MAPE). Google search trends evinced moderate positive correlations to the HFMD cases (r_0–6weeks_: 0.47–0.56), with temperature revealing weaker positive correlations (r_0–3weeks_: 0.17–0.22), with the association being most intense at 0–1 weeks. The SARIMA model, with regressors of mean temperature at lag 0 and Google search trends at lag 1, was the best-performing model. It provided the most stable predictions across the four-week period and produced the most accurate predictions two weeks in advance (RMSE = 18.77, MAPE = 0.242). Trajectorial forecasting oscillations of the model are stable up to four weeks in advance, with accuracy being the highest two weeks prior, suggesting its possible usefulness in outbreak preparedness.

## 1. Introduction

Hand, foot, and mouth disease (HFMD) is a viral-mediated infectious illness of increasing public health importance over the last quarter century caused primarily by the viral pathogens enterovirus A71 (EV-71) and coxsackie A16 (CVA-16) [1,2]. It is transmitted via bodily fluids, fecal and urine discharge, and fomites, causing a mostly mild clinical illness three to six days post-incubation. This clinical illness is characterized by ulcers of the mouth and vesicular lesions of the hand and foot, with or without a febrile phase [2]. Severe disease is rare, but can manifest as brainstem encephalitis, meningitis, acute flaccid paralysis, and neurogenic pulmonary edema. These can result in motor or cognitive sequelae or even death. Severe disease is mediated primarily by the EV71 virus [2,3]. Increasing epidemic potential has been witnessed in almost all of Asia [3].

A broader view of transmission dynamics suggests the possibility of ecological precipitants, such as temperature, humidity, and precipitation [3]. These environmental drivers have been widely studied in Asia and point towards varying degrees of association between weather and the incidence of HFMD [4,5,6,7,8]. Climate change, globalization, and rapid urbanization also seem to be linked to the transmission of disease [3]. Further warming of the earth of up to another 0.5 °C is expected in the next three decades due to climate change, and therefore, HFMD transmission may very well intensify [9]. Human internet search behavior may also predict the rise and fall of HFMD cases, as seen in a modeling exercise in China [10].

Public health control measures remain reactive, revolving primarily around outbreak control using measures such as quarantine and isolation of cases, closure of high-risk premises, and increasing public awareness of hygiene. The presence of preventive action remains limited [2]. Prevention can certainly alter the public health management of HFMD and, to that end, reduce the burden associated with HFMD. The shift to prevention via the modeling of HFMD has begun to gain pace, with models to explain transmission dynamics, predict disease magnitude and impact, and even quantify disease burden in the region [1,11,12].

The authors here aimed to study the association of HFMD with meteorological predictors, such as temperature, rainfall, humidity, and internet search patterns. These insights would then be used to develop a forecasting model that could inform preventive strategies to reduce the disease burden of HFMD.

## 2. Materials and Methods

### 2.1. Study Area

The Malaysian state of Sabah is located on the island of Borneo. It has a landmass of 739,040 km, with a rural and urban population mix totaling 3.9 million Malaysian nationals and approximately 800,000 to 1.4 million illegal immigrants [13,14]. The state is highly connected to the internet, with the third highest proportion of Malaysian internet users coming from Sabah [15]. The state experiences a tropical rainforest climate, with a mean temperature of 26.9 °C and a mean rainfall of 232.3 mm^3^. It rains throughout the year. The highest rainfall is observed between May–December, and the driest month is February. The warmest month is May, and the coolest is January, with an average temperature variation of 1.5 °C.

### 2.2. Data Sources

In Malaysia, HFMD is a notifiable infectious disease, as declared by law [16]. Notifications are registered on the Malaysian National e-Surveillance platform [17]. Data on HFMD cases and their sociodemographic characteristics were acquired from this platform via the Ministry of Health of Malaysia. Mid-year population data was collated from the Department of Statistics of Malaysia. All meteorological data were obtained from the Sabah Meteorological Department via its meteorological stations located across the state. The variables provided by the department included mean temperature (°C), maximum temperature (°C), minimum temperature (°C), mean relative humidity, and mean rainfall (mm^3^). All Google trends data are available as open source data from the Google trends database.

### 2.3. Data Analysis

All data were computed into weekly averages. Data was divided into a 7-year training set of 364 weeks and a 2-year testing set of 104 weeks. Demographic features of HFMD cases in Sabah were described. The spatial distribution of incidences was estimated and visualized across the study period. Seasonal and trend decomposition using Loess (STL) was then used to isolate signals of trend, seasonality, and ‘white noise’ from the time series of HFMD cases over the study period [18,19]. Cross-correlation function (CCF) tests, auto-correlation function (ACF) tests and partial auto-correlation function (PACF) tests were used to establish correlations between the HFMD cases and variables of temperature, rainfall, humidity, as well as search queries and their lagged terms [12]. A seasonal autoregressive integrated moving average (SARIMA) model was developed due to its flexibility in allowing for the control of the autocorrelation terms. In building the SARIMA model, the stationarity of the data was first assessed, as the presence of seasonality and the trend in a data series would affect the stationarity of said series. Transformation, in this case, the use of the differencing, was utilized in overcoming stationarity and developing the (D, d) terms to be integrated as a parameter in the SARIMA model. The second stage of model development involved the integration of the ACF-derived autoregressive (AR)—(P, p) processes and the PACF-derived moving average (MA)—(Q, q) processes, coalescing them into the functions (P, D, Q) (p, d, q) as seasonal and non-seasonal parameters of autoregression into the SARIMA model, respectively. These were tested iteratively for fit utilizing the Akaike Information Criterion (AIC) and log-likelihood, whereby a smaller value indicates a greater fit. A Ljung–Box test was used to check the ACF and PACF of the residuals and the normality of the residuals produced by the SARIMA to confirm model fit, where a significant test would be statistically different from zero. Upon selection of the best fitting SARIMA model, external regressors that were found to be significantly associated with HFMD cases in the correlation tests were iteratively integrated into the SARIMA with an external regressors model in a stepwise and forward selection method, guided by the same model fit metrics of AIC and log-likelihood. The final model is given by:

$$Y_{t}=\frac{{Ø}_{q}{\left(B\right)}_{Q}(B^{S})a_{t}}{{Ø}_{p}{\left(B\right)}_{P}(B^{S}){\left(1-B\right)}^{d}{\left(1-B^{S}\right)}^{D}}+X$$

where,

$${Ø}_{p}(B)=\;\mathrm{autoregressive}\;\left(\mathrm{AR}\right)\;\mathrm{operator},$$

$${Ø}_{q}(B)=\;\mathrm{non}-\mathrm{seasonal}\;\mathrm{moving}\;\mathrm{average}\;\left(\mathrm{MA}\right)\mathrm{operator},\;$$

$$\Theta_{P}(B^{S})=\;\mathrm{seasonal}\;\mathrm{AR}\;\mathrm{operator}$$

$$\Theta_{Q}(B^{S})=\;\mathrm{seasonal}\;\mathrm{MA}\;\mathrm{operator},$$

$${\left(1-B\right)}^{d}=\;\mathrm{non}-\mathrm{seasonal}\;\mathrm{difference}\;\mathrm{component},$$

$${\left(1-B^{S}\right)}^{D}=\;\mathrm{seasonal}\;\mathrm{difference}\;\mathrm{component},$$

X= independent variable defined here as temperature, 

rainfall, humidity and google trends,

$$a_{t}=\;\mathrm{white}\;\mathrm{noise},Y_{t}=\;\mathrm{dependent}\;\mathrm{variable}$$

Multiple iterations of the above metrics determined the best-fitting model. The best-fitting model was then selected for the final stage of forecasting model development, producing predictions and validating those predictions using the test dataset. The outcomes of the validation were two metrics—the root-mean-square error (RMSE) and mean average percentage error (MAPE). The mean average error (MAE) and mean squared error (MSE) were also compared. A smaller MAPE and RMSE signify greater forecasting accuracy [19,20]. All analyses were carried out using R version 3.6.0 [21].

## 3. Results

Over the study period, 14,929 cases of HFMD were reported in Sabah. The mean age of the cases was three and a half years, with more than 85% being under five. Males reported HFMD symptoms 11% more frequently than females. Most collated data was also from Malaysian nationals, with only 2% of the reported cases being non-national individuals. Cases were reported predominantly among the two largest ethnicities in Sabah—the indigenous Sabahans and the Chinese—with more than 85% of the cases reported among these two groups. The urban centers of Kota Kinabalu, Sandakan, and Tawau are the three largest cities in Sabah, and together, they make up close to 40% of cases in Sabah, with the neighboring economic hubs of Penampang and Tuaran accounting for another 15% of cases. These demographic characteristics are detailed in Table 1 below.

**Figure 1:**
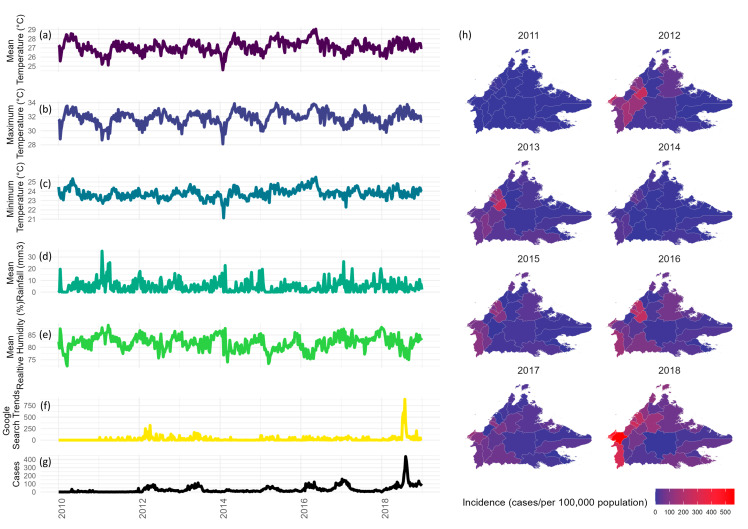
HFMD cases, temperature, rainfall, humidity, trends, and incidence in Sabah 2010–2018. (**a**) Mean temperature, (**b**) maximum temperature, (**c**) minimum temperature, (**d**) mean rainfall, (**e**) mean relative humidity, (**f**) Google search trends, (**g**) HFMD cases, and (**h**) spatial distribution of incidence.

Cases exhibit bi-modal seasonality with larger, more sustained transmission between February and July, followed by a much shorter and smaller peak between October and November of each year. Certain years record larger surges in case magnitude, although a general increasing trend is observed across the study period. HFMD cases are mostly observed after an increase in mean, maximum, and minimum temperatures above 27 °C, 30 °C, and 23 °C, respectively. A decrease in rainfall below 10–15 mm^3^ appears to trigger a rise in HFMD cases. Reductions in humidity below 75% trigger an increased number of HFMD cases. Meteorologic trends preceding cases are inconsistent. Increases in Google search trends consistently precede increases in HFMD cases. Incidences appear to be on an upwards trend across the study period, with a mean incidence of 51.4 cases per 100,000 people (range: 5.2–116.6). Higher incidences are recorded in larger, more populous districts, although all districts report a higher number of cases across time. These are visualized in Figure 1 and further explored using boxplots in Figures S1–S6.

The Google search trends variable proved to be positively and moderately correlated with HFMD cases, with the strongest correlation being at a lag of 1 week prior to cases (r = 0.52, *p* < 0.01), with the strength of correlation decreasing across time. The minimum, mean, and maximum temperatures were also calculated to have weak, but positive correlations with HFMD cases. Of these, the mean temperature at lag 0 recorded the highest levels of correlation with HFMD cases (r = 0.22, *p* = 0.01). As observed in Google search trends, the effects of temperature on HFMD cases also decreased over time. Intriguingly, mean relative humidity and mean rainfall recorded weak negative correlations with HFMD cases, with the correlation being the strongest at a mean relative humidity of lag 0 (r = −0.15, *p*-value = 0.01) and a mean rainfall of lag 0 (r = −0.11, *p* = 0.02). A cross-correlation table is provided in Table S1.

**Figure 2:**
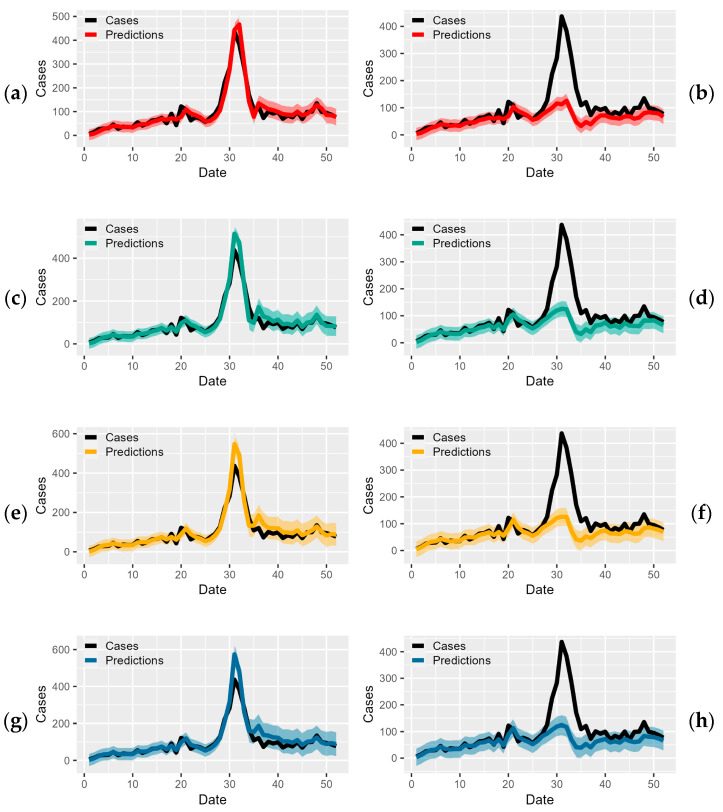
Predictions of HFMD compared to actual HFMD cases; (**a**) actual against 1-week advance predictions of HFMD cases, 2017–2018, using SARIMA model, (**b**) actual against 1-week advance predictions of HFMD cases, 2017–2018, using SARIMA with external regressors model, (**c**) actual against 2-week advance predictions of HFMD cases, 2017–2018, using SARIMA model, (**d**) actual against 2-week advance predictions of HFMD cases, 2017–2018, using SARIMA with external regressors model, (**e**) actual against 3-week advance predictions of HFMD cases, 2017–2018, using SARIMA model, (**f**) actual against 3-week advance predictions of HFMD cases, 2017–2018, using SARIMA with external regressors model, (**g**) actual against 4-week advance predictions of HFMD cases, 2017–2018, using SARIMA model, and (**h**) actual against 4-week advance predictions of HFMD cases, 2017–2018, using SARIMA with external regressors model.

A single order of differentiation produced a stationary ADF (DF = −6.54, *p* = 0.01) and KPSS (KPSS = 0.035, *p* = 0.1). The (P, D, Q) (p,d,q) parameter space was iteratively explored. The best-fitting model reported an AIC of 3356.45 and a log-likelihood score of −1672.23. A Ljung–Box test was carried out, which reported the independence of the residuals (*p* = 0.29), signifying no remaining autocorrelations. Variables were then integrated into the model, employing a stepwise and forward-selection method. The best model that balanced parsimony and performance measured by the AIC and log-likelihood metrics was the model with two variables of mean temp at lag 0 and Google search trends at lag 1. The results of validating these two models are highlighted in Table 2. The SARIMA model, with regressors of mean temperature at lag 0 and Google search trends at lag 1, was the best performing model, exhibiting the most stability across the four-week prediction period and producing the most accurate predictions two weeks in advance (MAE = 12.28, MAPE = 0.242, MSE = 422.09, RMSE = 18.77). Both models, however, produced forecasts that decayed across longer periods, with predictions at week 1 being better than those at weeks 2, −3, and −4 (Figure 2).

## 4. Discussion

HFMD transmission in Sabah typically occurs between February and July, peaking in April or May, with a subsequent ancillary epidemic in October or November. However, there remains the potential for sporadic epidemics all year long. This pattern of bimodal transmission is reported across the literature in tropical and subtropical regions [3,8,22,23].

We found that the SARIMA with external regressors of mean temperature at lag 0 and Google search trends at lag 1 predicting two weeks into the future produced the most accurate forecasts over the two-year test period (MAE = 12.28, MAPE = 0.28, MSE = 352.29, RMSE = 18.77). The forecast accuracy is comparable to those produced by the SARIMA model with an external regressor of temperature at a lag of two weeks in Zhengzhou, China (RMSE = 35.2), a meta-learning model utilizing the Baidu Index in Guangdong, China (RMSE = 34.01), a SARIMA model in Shezuan, China (MAPE = 0.28–0.37), an ARIMA model in Sichuan, China (MAPE = 0.15), another ARIMA model in Changsha, China (RMSE = 8.29), and finally, an ARIMAX model utilizing external regressors of the Baidu Index and temperature at lags 0 in Guangdong, China (MAPE = 1.02) [10,12,24,25,26,27]. In comparing the metrics of validation of the HFMD forecasting models available in the literature (MAPE= 0.15–1.30, RMSE = 8.29–35.2) to the models developed utilizing the data from Sabah (MAPE = 0.16–0.28, RMSE = 18.77–30.49), the forecasting models developed here are at least equivalent, if not better than most models currently available in the contemporary literature. Models like the one developed here can benefit public disease prevention by priming risk communication channels in advance, optimizing resource allocation, and utilizing capacity-building planning.

Despite multiple predictors tested as significant in the correlation tests, only temperature and Google trends statistically improved model forecasts, as has also been demonstrated in several other regional studies [10,12,24]. Despite the biological plausibility of the association between humidity, rainfall, and HFMD cases, in model fitting, the effect that these two predictors had on HFMD forecasting was low. Temperature and HFMD cases in Sabah exhibited the most significant relationships at lags of 0 (r = 0.22). This positive relationship has not only been replicated in the more temperate countries of Japan, China, Hong Kong, and Taiwan, but also in the more tropical and sub-tropical regions of Thailand, Vietnam, Singapore, and other parts of Malaysia [28,29,30,31,32,33,34,35]. Studies have speculated that higher temperatures are likely to change social behaviors—with warmer weather encouraging social interactions, such as children playing outside in a neighborhood, that increase the likelihood of transmission events [36]. Google search trends reported positive and moderate associations at a lag of 1 week (r = 0.56). Several Chinese studies have examined the Chinese equivalent of Google search trends—the Baidu index, and its association with HFMD cases. These studies have found that HFMD cases are highly correlated with the Baidu index (r = 0.68–0.95) and, as such, it was utilized in the forecasting models [24,25,37].

In Sabah, denser clustering of cases can be visualized in the more urban, social, and economic hubs such as Kota Kinabalu, Sandakan, and Tawau. As has been observed in several other studies, urban settings are postulated to increase the risk of transmission compared to rural settings [28,29,30]. Urban clustering is likely due to factors such as higher population density, higher levels of preschool attendance, the use of communal toilets, poor hygiene practices due to overcrowding, and even outside consumption of food [31,32,33,34]. Intriguingly, several rural districts across the western border of Sabah reported a much higher incidence rate as opposed to other similarly “rural” districts across its eastern border. While it cannot be confirmed as yet, travelling waves of contagion between the neighboring state of Sarawak into Sabah is a possibility, as has been observed in diseases such as influenza, measles, and dengue [35,36,37]. There are several important limitations of this study. The study design, particularly in the first stage of analysis, is prone to ecological fallacy, as generalizations from population-based studies on individuals may be prone to inferential error, spurious findings, or unmeasured confounding factors [5]. Several important predictors of HFMD transmission, such as family dynamics and traveling waves, along with other ecological predictors, such as wind speed, PM10, vegetation index, socioeconomic status, and ultraviolet radiation, as well as geographical settings, such as rural or urban, and the timing of school or public holidays, were not considered in this study. Similarly, the long latency of HFMD viruses within the stool of infected individuals creates long latency in carriers of up to 62 days, and with asymptomatic carrier rates being as high as 70% in certain study localities, these create an iceberg phenomenon that is likely to challenge the precision and interpretation of the lag effects of weather variables [3,8]. Google search trends also carry a certain inherent weakness arising from the selection of keywords in developing a search strategy to be utilized in forecasting. Nonetheless, the real-world implementation of models such as this should be carried out in assessing their real-world effectiveness and identifying specific areas for improvement. Additionally, model development by iterating different methods and data should be continued in the future.

## 5. Conclusions

Oscillations of HFMD epidemics in Sabah are bimodal and on an upward trend. Important predictors of HFMD in Sabah include temperature and Google search trends, which can be utilized to assist in outbreak preparedness and mitigation—essential stages in the day-to-day work of an epidemiological disease control division. The reinforcement of public health institutions in the preparedness and mitigative phase of outbreak management is urgent. A resilient public health system capable of foreseeing and tackling communicable disease threats far ahead of time remains an important goal.

## Figures and Tables

**Table 1:** Sociodemographic characteristics of HFMD cases in Sabah from 2010–2018.

		Overall (*n* = 14,929)
Age (mean (SD))		3.55 (5.24)
Age Group (%)	<1 year old	1385 (9.3)
	1–2 years old	6779 (45.4)
	3–5 years old	4619 (30.9)
	6–14 years old	1713 (11.5)
	>15 years old	433 (2.9)
Gender (%)	Female	6605 (44.2)
	Male	8324 (55.8)
Nationality (%)	Malaysian	14,682 (98.3)
	Non-Malaysian	247 (1.7)
Ethnicity (%)	Chinese	2308 (15.5)
	Indian	37 (0.2)
	Malaysian	1079 (7.2)
	Indigenous Sabahan	10,581 (70.9)
	Indigenous Sarawakian	143 (1.0)
	Others	524 (3.5)
	No information	257 (1.7)
Location (%)	Rural	4805 (32.2)
	Urban	10,124 (67.8)

Note: Values are in absolute number (%), unless otherwise stated.

**Table 2:** Validation of selected SARIMA and SARIMA with external regressors model.

Period (Weeks Ahead)	Method	MAE	MAPE	MSE	RMSE
1	SARIMA	12.91	0.16	395.76	19.89
	SARIMA with External Regressors	14.83	0.28	544.74	23.34
2	SARIMA	13.66	0.15	548.30	23.46
	SARIMA with External Regressors	12.28	0.26	352.29	18.77
3	SARIMA	17.26	0.18	845.44	29.08
	SARIMA with External Regressors	13.45	0.242	422.09	20.55
4	SARIMA	16.91	0.17	929.79	30.49
	SARIMA with External Regressors	13.46	0.25	404.36	20.11

## Data Availability

All data used within the study belongs to the Ministry of Health, Malaysia and the Department of Meteorology, Malaysia. Data are available from respective ministries on reasonable request.

## Supplementary Materials

The following supporting information can be downloaded at: https://www.mdpi.com/article/10.3390/ijerph192416880/s1, Table S1: Spearman’s rank correlation between HFMD cases and independent variables. Figures S1–S6: Boxplots of HFMD cases, along with meteorologic and Google search trends.
