# Supplementary material for: Forecasting HFMD Cases Using Weather Variables and Google Search Queries in Sabah, Malaysia
Source: Int J Environ Res Public Health. 2022 Dec 15;19(24):16880. doi: 10.3390/ijerph192416880 (PMC9779090; doi:10.3390/ijerph192416880)
Supplement: Supplementary file 1 [file ijerph-19-16880-s001.zip › ijerph-1921018-supplementary.docx]

Supplementary materials

Table S1: Spearman rank correlation between HFMD Cases and Independent Variables

|  | r | | | | | |
| --- | --- | --- | --- | --- | --- | --- |
|  | Mean Temperature | Maximum Temperature | Minimum Temperature | Mean Relative Humidity | Mean Rainfall | Google Search Trends |
| Lag 0 | 0·22* | 0·17* | 0·19* | -0·15* | -0·11* | 0·53* |
| Lag 1 | 0·20* | 0·16* | 0·18* | -0·14* | -0·10* | 0·56* |
| Lag 2 | 0·17* | 0·14* | 0·17* | -0·14* | -0·09 | 0·52* |
| Lag 3 | 0·16* | 0·12* | 0·16* | -0·14* | -0·09 | 0·50* |
| Lag 4 | 0·15* | 0·11* | 0·16* | -0·13* | -0·08 | 0·49* |
| Lag 5 | 0·15* | 0·11* | 0·16* | -0·13* | -0·05 | 0·49* |
| Lag 6 | 0·15* | 0·09 | 0·19* | -0·13* | -0·07 | 0·47* |

*p <0·05


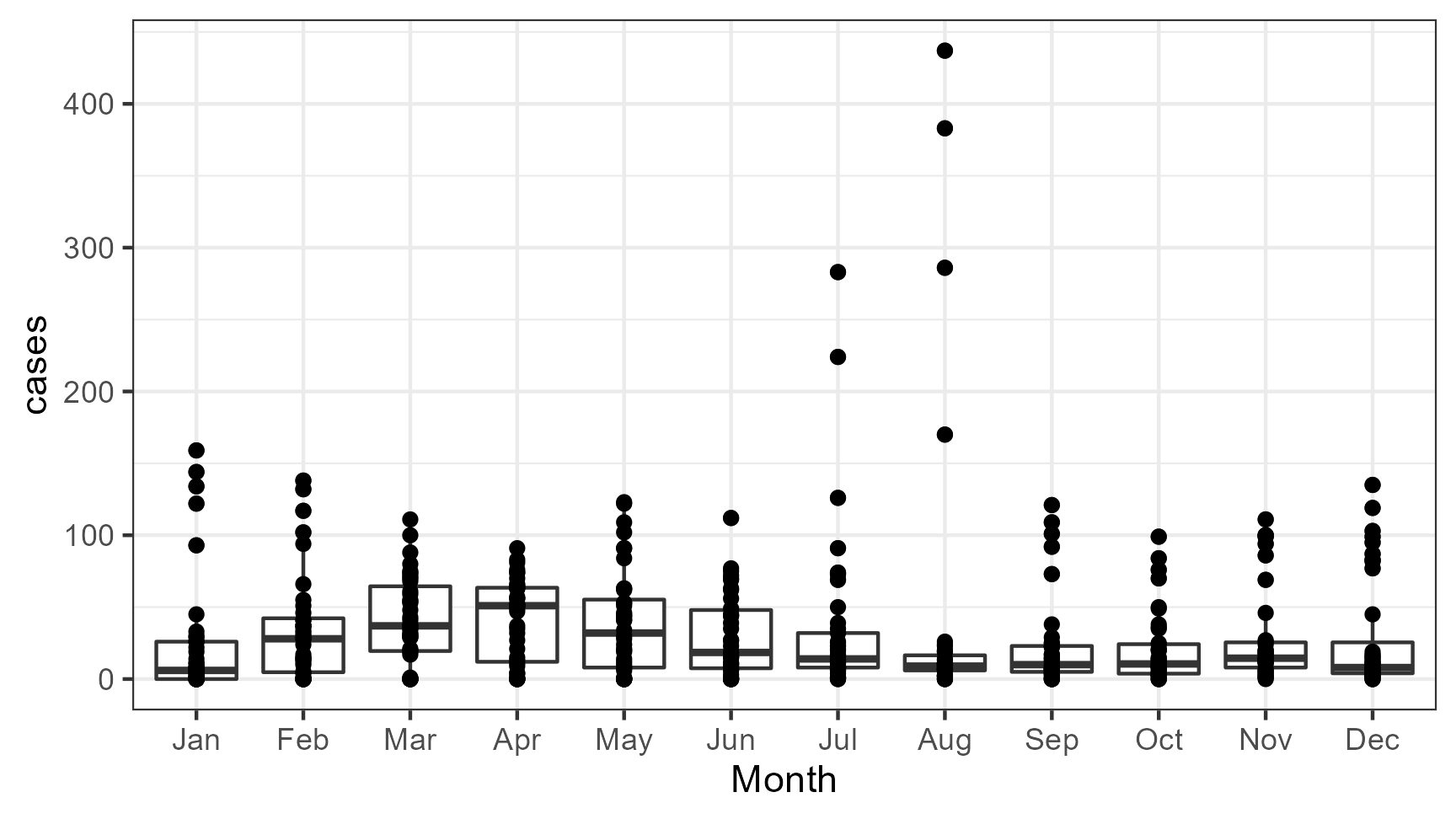


Figure S1: Boxplot of HFMD cases in Sabah between January 2010 and December 2018


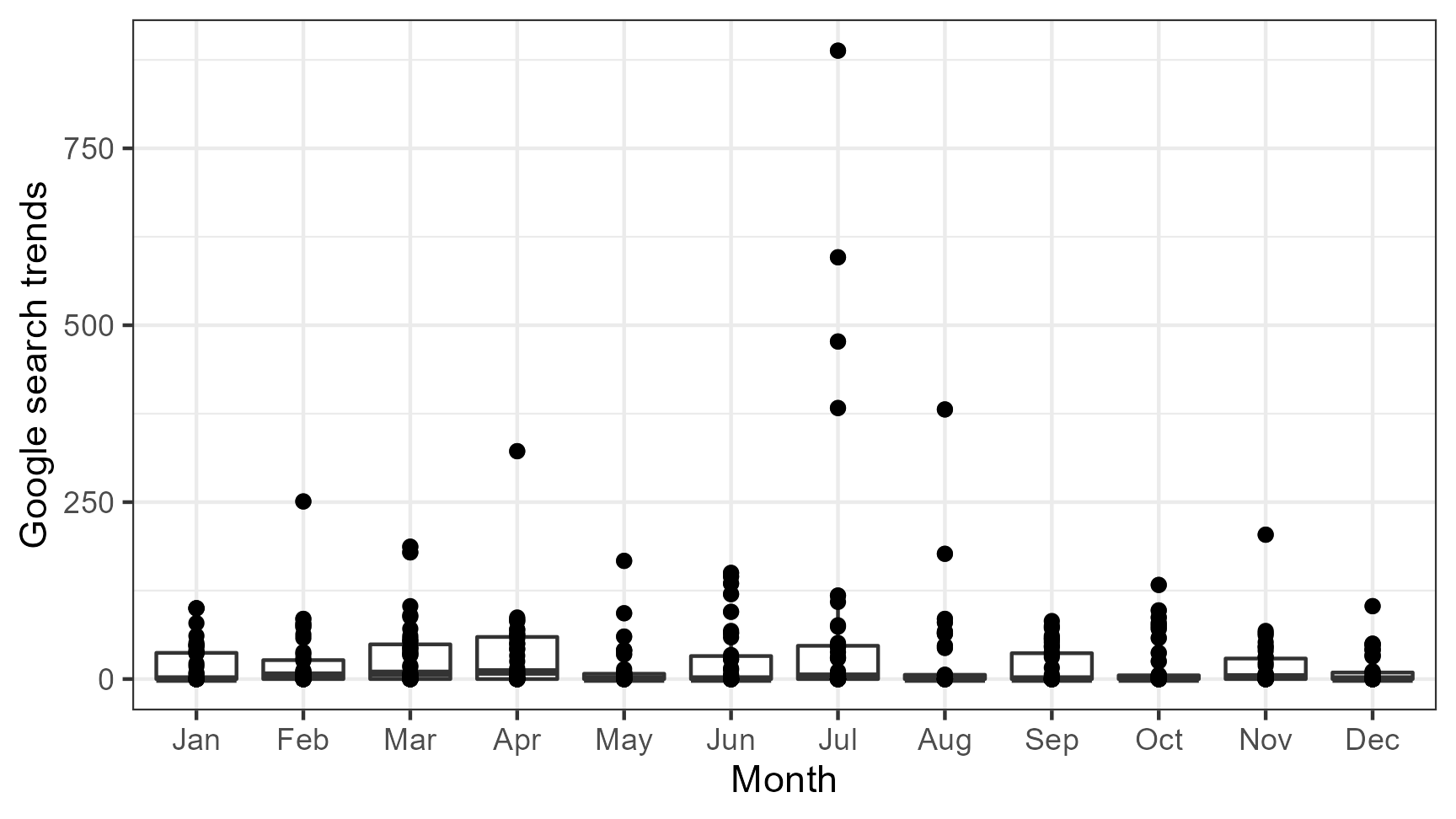


Figure S2: Boxplot of Google search trends in Sabah between January 2010 and December 2018


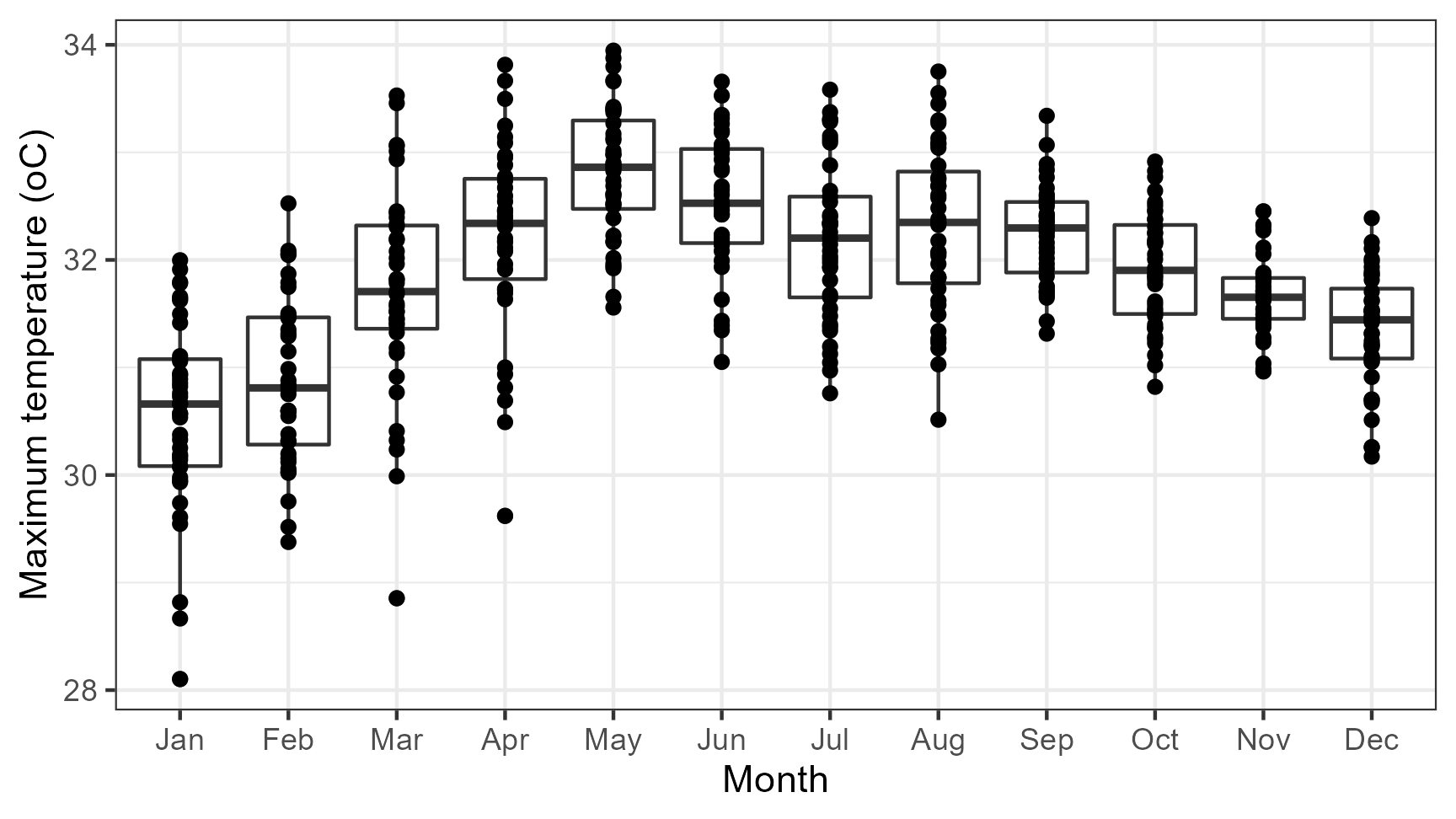


Figure S3: Boxplot of maximum temperature in Sabah between January 2010 and December 2018


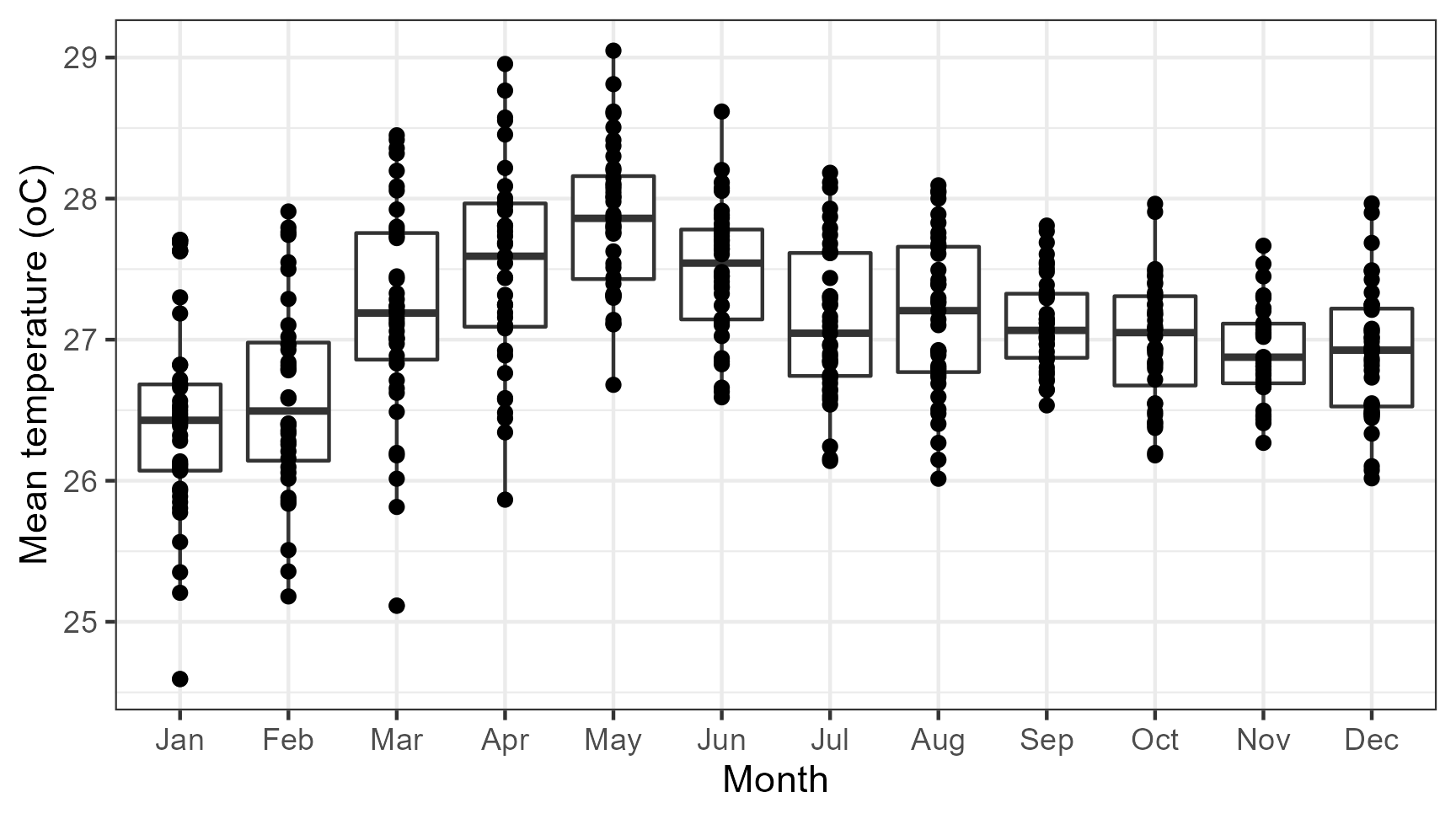


Figure S4: Boxplot of mean temperature in Sabah between January 2010 and December 2018


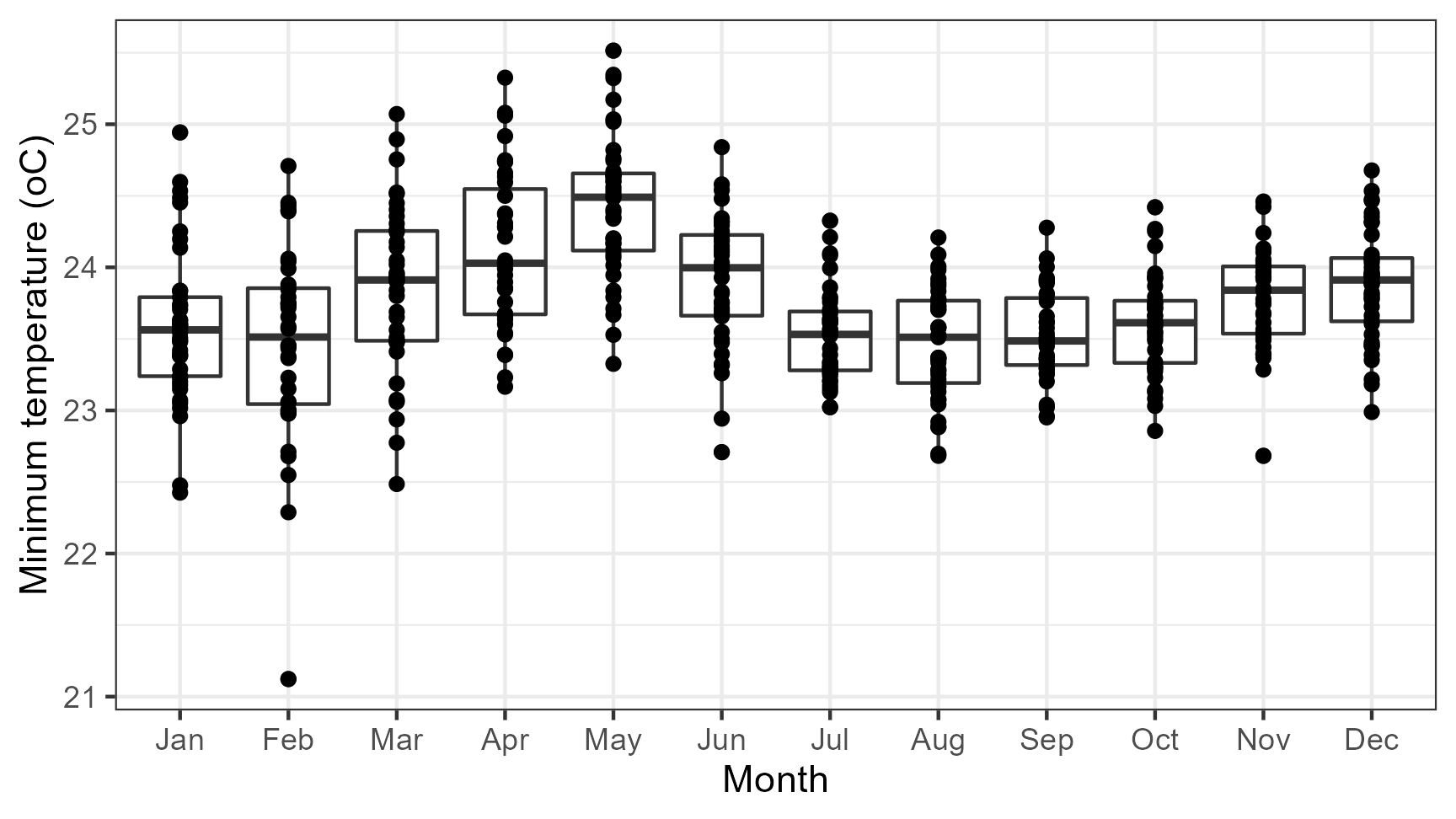


Figure S5: Boxplot of minimum temperature in Sabah between January 2010 and December 2018


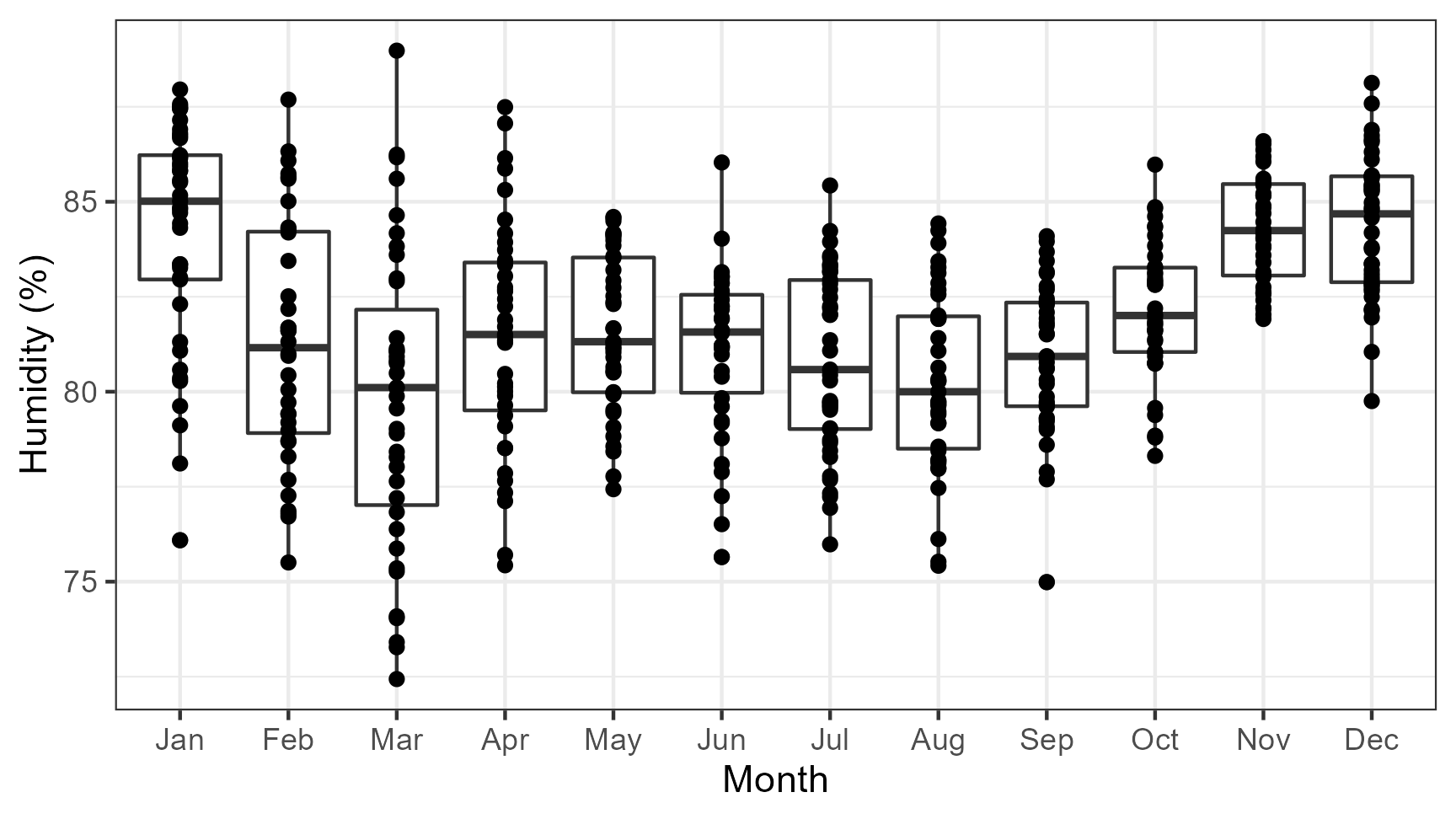


Figure S6: Boxplot of mean relative humidity in Sabah between January 2010 and December 2018


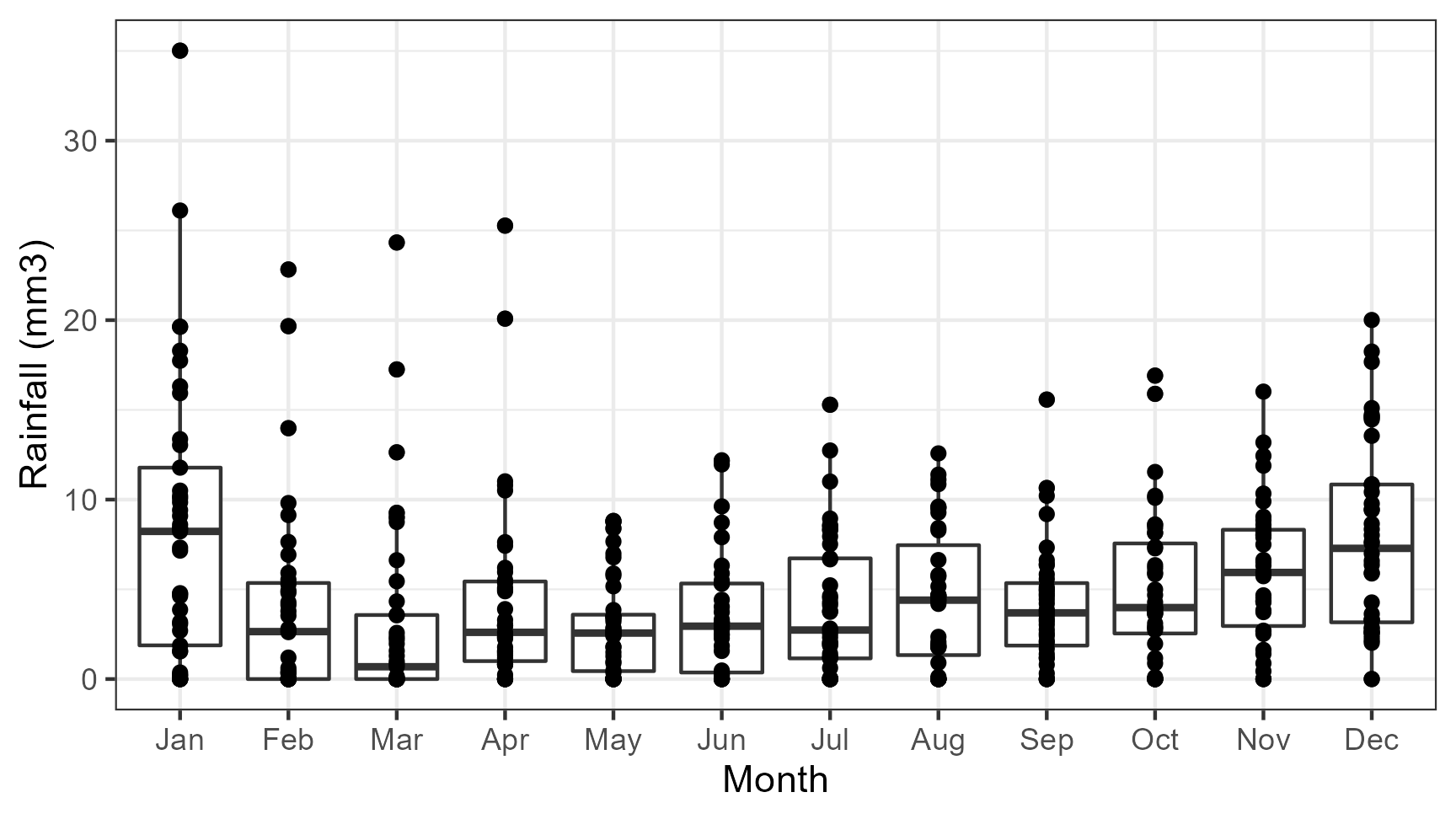


Figure S7: Boxplot of mean rainfall in Sabah between January 2010 and December 2018
